# An algorithm for automatically generating gas, bone and foreign body visualizations from postmortem computed tomography data

**DOI:** 10.1007/s12024-021-00363-3

**Published:** 2021-04-27

**Authors:** Lars C. Ebert, Dilan Seckiner, Till Sieberth, Michael J. Thali, Sabine Franckenberg

**Affiliations:** 1 3D Center Zurich, Institute of Forensic Medicine Zurich, University of Zurich, Winterthurerstrasse 190/52 CH-8052, Zurich, Switzerland; 2 Institute for Diagnostic and Interventional Radiology, University Hospital Zurich, Zurich, Switzerland

**Keywords:** Virtopsy, PMCT, Visualization, Maximum intensity projection, Minimum intensity projection

## Abstract

Post mortem computed tomography (PMCT) can aid in localizing foreign bodies, bone fractures, and gas accumulations. The visualization of these findings play an important role in the communication of radiological findings. In this article, we present an algorithm for automated visualization of gas distributions on PMCT image data of the thorax and abdomen. The algorithm uses a combination of region growing segmentation and layering of different visualization methods to automatically generate overview images that depict radiopaque foreign bodies, bones and gas distributions in one image. The presented method was tested on 955 PMCT scans of the thorax and abdomen. The algorithm managed to generate useful images for all cases, visualizing foreign bodies as well as gas distribution. The most interesting cases are presented in this article. While this type of visualization cannot replace a real radiological analysis of the image data, it can provide a quick overview for briefings and image reports.

## Introduction

Postmortem computed tomography (PMCT) is an image modality routinely used at present within many western forensic institutes as a valuable adjunct in forensic postmortem examinations. It allows for noninvasive first insights and diagnosis of almost all forensic relevant findings, sometimes even demonstrating findings that may have been missed at autopsy [1]. PMCT not only aids in the location of projectiles and in determining the course of a projectile path but also in precisely assessing fractures, soft tissue lesions and many other injuries [2]. In terms of intracorporeal gas, it is possible to differentiate between (physiological) decompositional gas, (vital) emphysema and (potentially lethal) gas embolism [3]. It can also contribute to the identification of unknown persons via medical implants and devices or dental records [4]. Additionally, the stored CT data can be evaluated repeatedly (in contrast to autopsy) with different objectives that may arise during forensic investigations even days and/or months after the initial event [5].

The visualization of PMCT data plays an important role in the communication of radiological findings to state attorneys, lawyers and judges [6]. Unlike radiologists who are used to working on grayscale 2D slices and multiplanar reconstructions (MPRs), state attorneys prefer a highlighted depiction of the findings with color-coded images or even 3D visualizations, which are usually created manually. Manual visualizations are of different complexity and can potentially be quite time-consuming. They involve a variety of different techniques, depending on the information that is to be highlighted. High-density materials, such as bone or foreign bodies, can be visualized best by using a maximum intensity projection (MIP) [7]. A MIP converts a 3D dataset into a 2D image by projecting the voxels with the highest density onto an image plane. To visualize gas, which has a low X-ray density, the opposite of a MIP, a minimum intensity projection (minIP) can be calculated, which projects voxels with the lowest density onto the image plane. Concurrently, in clinical radiology, increasingly more software applications are being created for automated visualizations in cardiac imaging. Except for predefined 3D-volume overview renderings for the assessment of bone fractures and specialized tools for rib and skull unfolding, there is hardly any fully automated visualization in postmortem radiology [8–10].

In this article, we present an algorithm for automated visualization of PMCT data of the thorax and abdomen resulting in one 2D projection image of the body simultaneously showing intracorporeal gas, radiopaque foreign bodies (medical devices, implants, projectiles, etc.) and bones (anatomical orientation, gross fracture patterns).

## Materials and methods

### PMCT and case collective

We retrospectively extracted all consecutive primary autopsy cases from the case archive of the Zurich Institute of Forensic Medicine from January 2017 to June 2019. No exclusion criteria were employed. This resulted in 955 cases in total. A total of 612 of these cases were male, and 343 cases were female, with an age between newborn and 100 years (average 54.2 years, standard deviation 22.4 years). For all cases, whole-body PMCT prior to autopsy was performed on a Siemens Somatom Definition Flash (Siemens Healthineers, Erlangen, Germany), with standard reconstructions of the head and thorax/abdomen. For our study, we exported the hard kernel reconstructions (B60) of the thorax and abdomen from the PMCT database for all cases, as the hard kernel allowed for the best depiction of bones, air and radiopaque material with the disadvantage of generating noisier images. For our experiments, we selected primarily thorax/abdomen reconstructions. Demographic data and case information were extracted from the forensic reports. From a prior study, the radiological alteration index (RAI) was available for all cases. The RAI is a means to quantify gas accumulations using a standardized scoring system [3].

### Software and algorithm

We developed a custom-made command line application to automatically convert thorax/abdomen PMCT scans into a single visualization highlighting gas distribution, bones and foreign bodies. The software was developed in C +  + using Visual Studio and based on the Visualization Toolkit (VTK) library version 8.2.0 and QT 5.14.1.

**Fig. 1:**
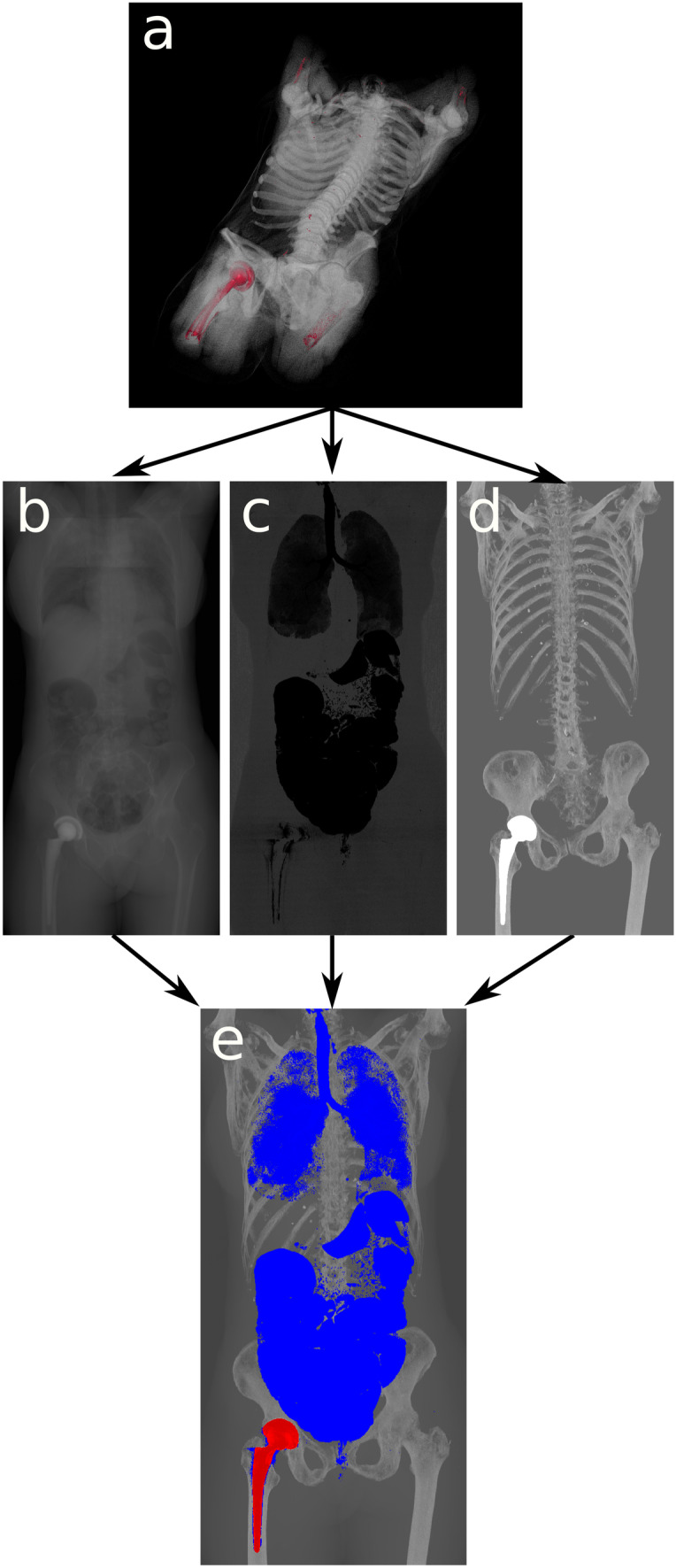
Workflow of the algorithm used. **a** Original PMCT volumetric dataset. **b** Averaged projection of the PMCT dataset resembling a radiograph. **c** minIP of the inside of the body. **d** MIP projection of the dataset. **e** Image combining b,c and d into one overview image

**Fig. 2:**
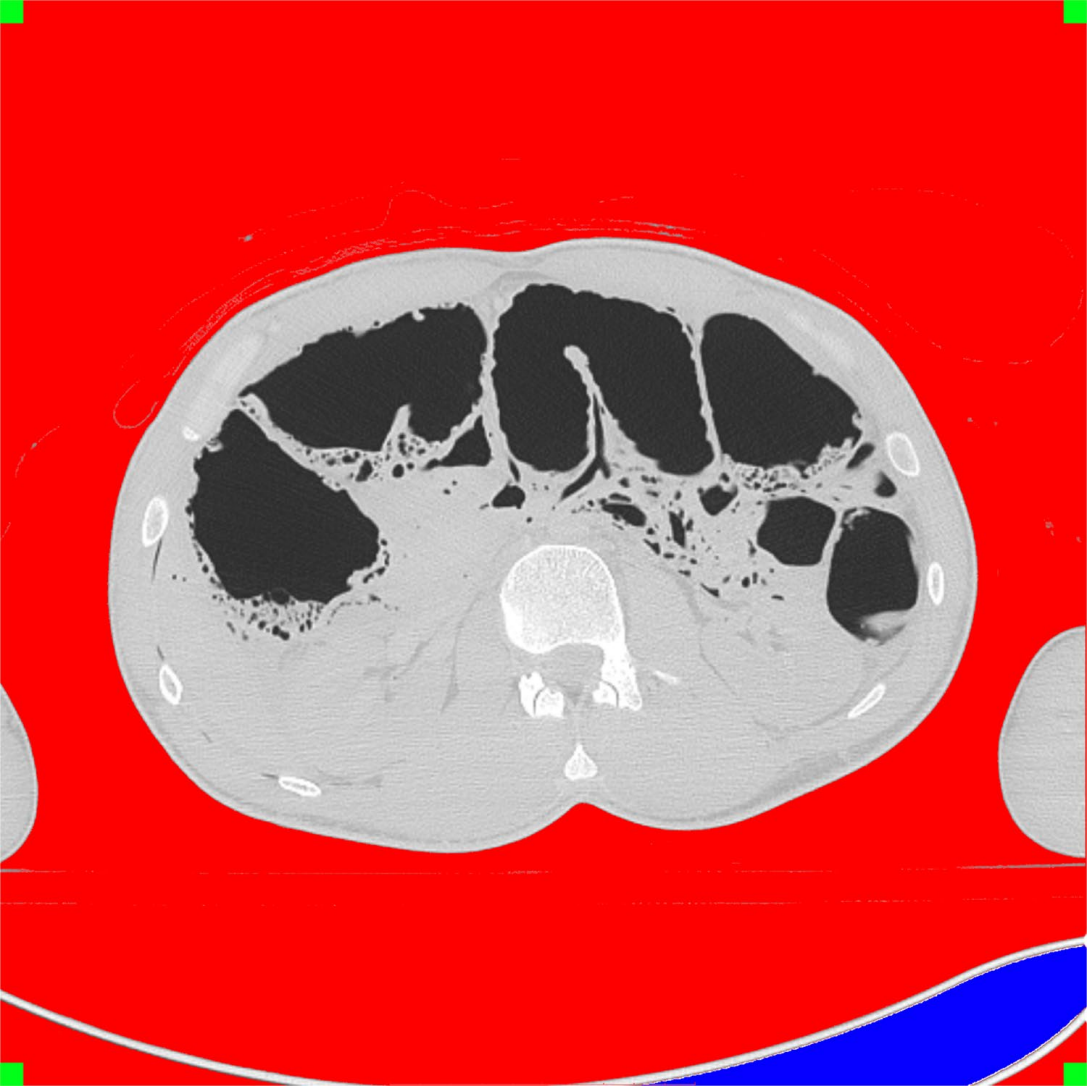
Segmented slice of a PMCT scan. When taking the corners as seed points for region growing, air outside the body is marked (red), while air inside the body is not affected. Parts of the CT table are air filled and might not be reached from the corner points (blue)

The algorithm works in four steps (Fig. 1). First, the dataset is loaded. Second, it is necessary to distinguish low-density voxels from inside the body from the air around the body, including body bags. A region growing algorithm is conducted to remove all voxels outside of the body with a Hounsfield unit (HU) of -200 or less. For this, all eight corners of the CT volume are selected as seed points as these are always outside the body. The inside of our specific CT scanner uses a table that is hollow and filled with air that cannot be reached by only these seed points (Fig. 2). Therefore, the lower border of the CT table is detected and the first pixel with a HU lower than -200 is added to the list of seed points. This procedure is repeated for the left and right side of the CT table. In the third step, for both, the anterior–posterior and lateral views, three projections are created—a MIP, a selective minIP of the interior of the body and an average of the dataset. These three images are combined to generate the final image. The selective minIP ignores all voxels that have been selected by the region growing algorithm, thus only visualizing the gas distribution inside the body. The MIP is used to create a visual reference frame by highlighting the skeletal structures in the background. It is also the basis for identifying areas of high X-ray density caused by metal foreign bodies. The average projection is windowed (center: 500 width: 1000) to be able to add soft tissue references to the final image. In the final step, all images (MIP, minIP, average) are combined according to the following algorithm:

For all pixels in the final image:

- 1. The pixel in the final image is 0.7* HU of the MIP image pixel + 0.3 * HU of the average image pixel;
- 2. If the Hounsfield value on the pixel of the MIP is larger than 2800, the pixel in the final image is red;
- 3. If the Hounsfield value on the pixel of the MinIP is lower than -220, the pixel in the final image is blue.

The final image is exported as portable network graphics (png) file.

This procedure highlights high-density material in red and gas inside the body in blue and gives anatomical context in the form of visible bones and faint soft tissue. Lateral and anterior–posterior images were generated for each dataset.

After image generation, a board-certified radiologist visually inspected the images for visualization issues such as artifacts or incorrect visualizations and selected cases that highlighted what this type of visualization could be used for. As a preliminary test, we added examples for reconstructions of full body scans with extended field of view as well as head scans.

## Results

The algorithm managed to calculate useful overview images for all provided cases.

**Fig. 3:**
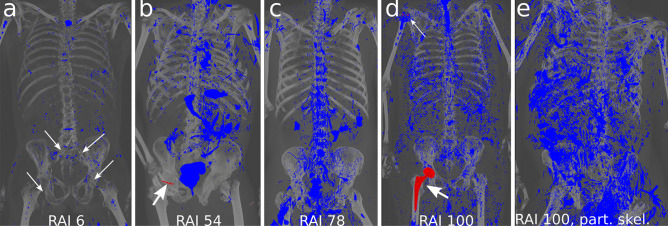
Visualizations at various stages of decomposition. **a** Minimal decomposition with advanced arteriosclerosis of the infrarenal aorta and the proximal femoral vessels (arrows). **b** Moderate signs of decomposition (RAI 54) and bone screw placement in the right femoral head (arrow). **c** Moderate to advanced signs of decomposition (RAI 78). **d** Advanced signs of decomposition (RAI 100), screws from osteosynthesis in the right proximal humerus (thin arrow), right hip prosthesis for potential ID (bold arrow). **e** Advanced decomposition with partial skeletonization (RAI 100)

For various advanced stages of decomposition (Fig. 3), our reconstructions allow for a quick initial assessment of the internal decomposition state.

**Fig. 4:**
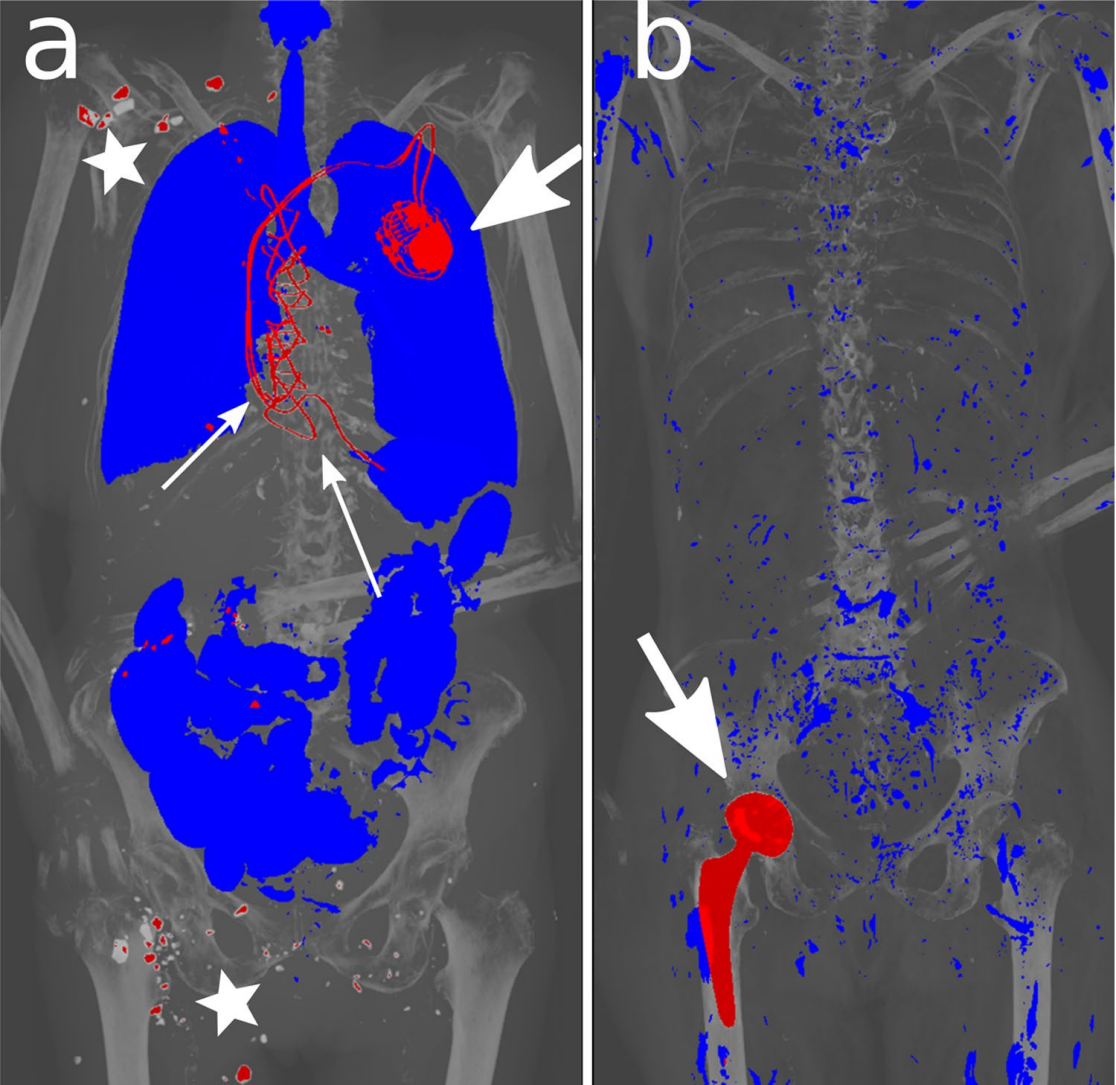
Two visualization examples of medical devices. **a** An 84-year-old female who died in an accident and was found outdoors. Pacemaker (bold arrow) with two electrodes, sternal cerclages (thin arrows) and small stones adhering to the skin (stars) are highlighted in red. **b** An 81-year-old female who died due to brain hemorrhage. Identification was performed by comparing the serial number of the hip implant (arrow) with the serial number of the surgical report of the suspected deceased

**Fig. 5:**
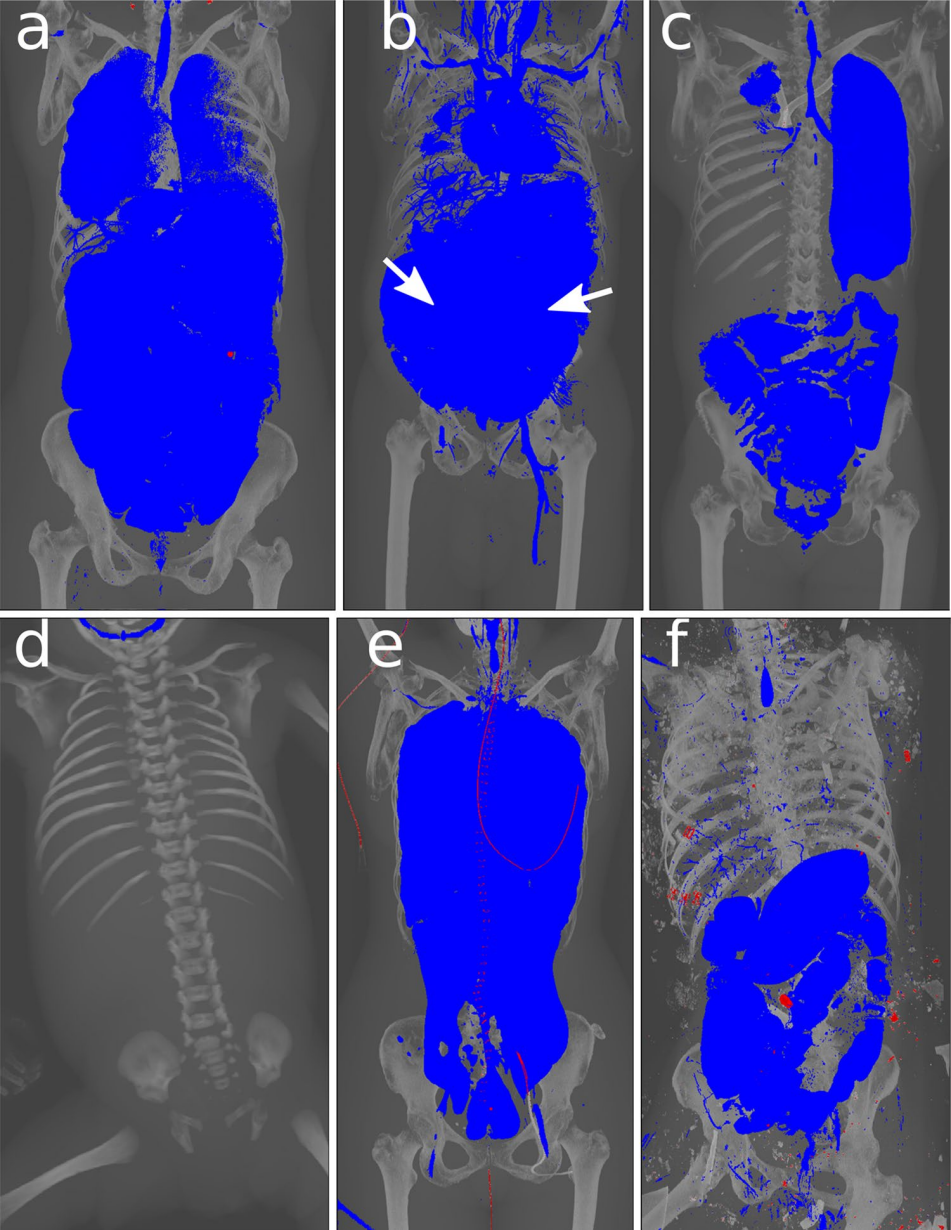
Various examples that highlight different findings. All findings were confirmed by autopsy. **a** Pneumonia with less gas in the left lung (arrows). **b** Excessive intra-abdominal gas (bold arrows) as an expression of more rapid and pronounced bacterial colonization due to colitis. **c** Near complete atelectasis of the right lung due to a carcinoma of the right upper lobe. **d** A female fetus (gestational age of 37 weeks) that died due to hypoxemia during birth. Due to a lack of respiration, the lung and abdomen contained no gas. **e** A 59-year-old female who died in an accident and had organs explanted for transplantation. **f** A 42-year-old female who died in a fire due to asphyxiation. Missing gas in the lungs corresponded to the fire-induced carbonization of the lung. Multiple foreign bodies from the site are visible

**Fig. 6:**
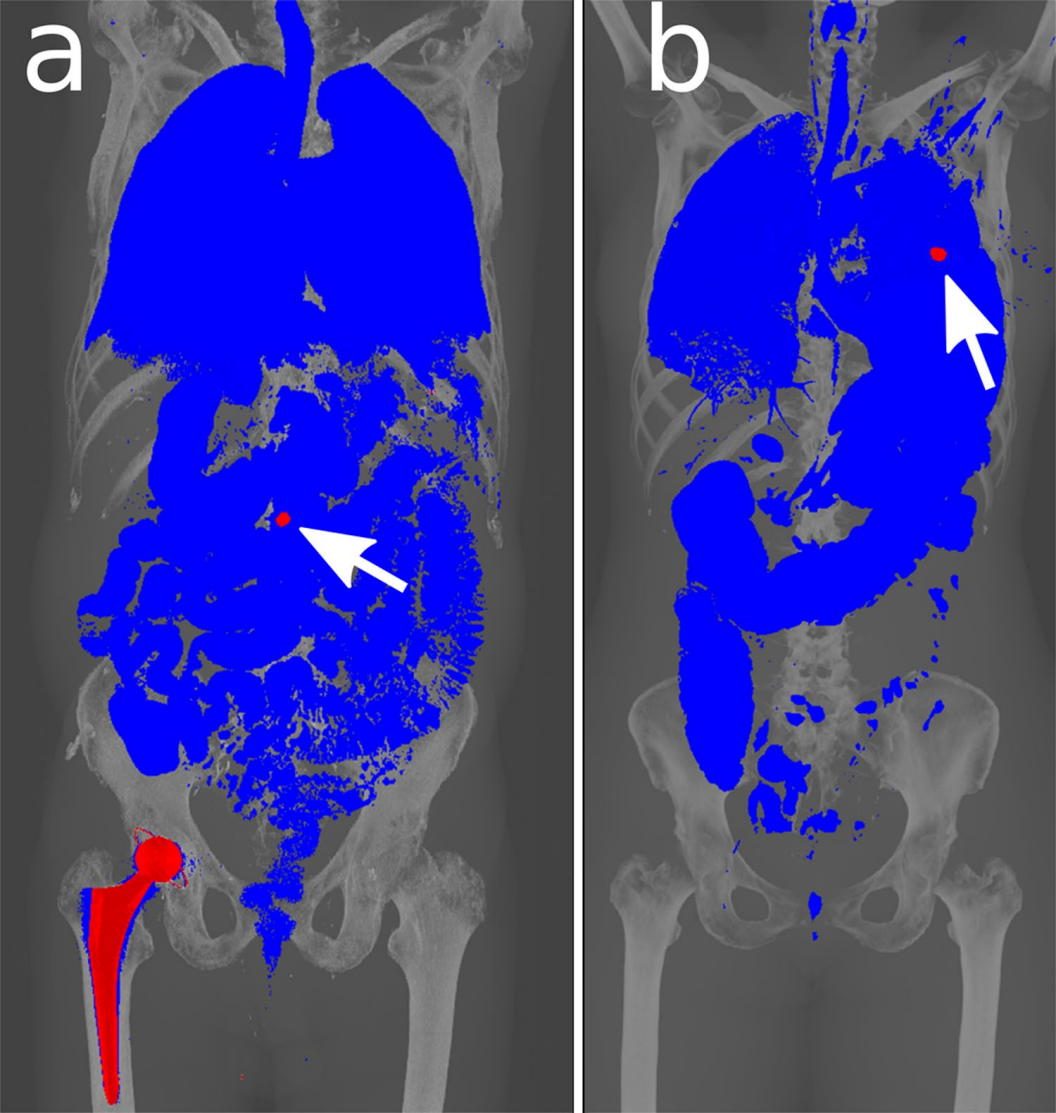
Two examples of gunshot wounds. **a** Projectile is marked with an arrow. Total hip replacement on the right side is visible. **b** Projectile in the left lung (arrow)

An exemplary selection of our cases, divided into “identification/medical devices”, “decomposition”, “gunshot” and “special cases”, is shown in Figs. 4, 5, 6. With most medical devices being radiopaque, such as pacemakers, osteosynthesis material and prostheses, they are perceived immediately in Fig. 4. This means that at first glance, potential identification material (via serial number of devices) can be detected.

In gunshot cases with the projectile remaining intracorporeal (Fig. 6), we are able to immediately determine its location.

In the special cases section (Fig. 5), we show a variety of particular findings that have an impressive corresponding pictorial equivalent in our reconstructions.

## Discussion

In this article, we present a new visualization technique for the generation of dedicated overview images from PMCT data. The presented technique compresses a 3D dataset into a 2D image, preserving the most relevant findings. The algorithm primarily allows for the immediate depiction of radiopaque medical devices, various advanced stages of decomposition, gross fracture patterns and projectiles. Beneath these elementary findings, we also discovered a variety of other findings with a striking corresponding pictorial equivalent, such as in cases of pneumonia, sepsis or thermal injury. In principle, similar reconstructions are also possible for full body scans and head scans.

We found that this algorithm works robustly for all PMCT thorax/abdomen volume data, regardless of cause and manner of death, and independent of the length of the postmortem interval and age of the deceased. In contrast to other visualization methods, such as the volume rendering technique [7] or cinematic rendering [11], this visualization is fully automatic, and no user interference such as thresholding, perspective, window or transfer function has to be defined.

Due to the software development libraries using VTK and QT, integration into the Syngo via Frontier platform is possible. This allows for the generation of overview images directly from the PACS graphical user interface (GUI), enabling an automatic creation of overview images from a PMCT scan without any additional expenditure of user time. Due to the simplicity of the algorithm, it can be adapted to other systems with a plugin interface, such as Horos (Horos Project, www.horosproject.org), relatively quickly.

In forensic daily routines, this visualization technique is not only applicable when initial PMCT findings are shown in the morning report. It is also convenient for image reports created for state attorneys, who prefer processed, color-coded radiological images for better understanding, which can be a time-consuming task if done manually. Automated visualizations such as the presented one can help overcome obstacles related to interpretation that may, for instance, be attributed to medical anatomy [6]. Furthermore, images such as these may make it easier to apply machine learning techniques to 3D datasets, i.e. for the automated estimation of the radiological alteration index (RAI) [12].

Generating these types of overview images presents a few limitations. While the algorithm itself works reliably for thorax/abdomen images, full body reconstructions can be problematic. First, if the oral cavity is open and the airways are uncompromised, there is a direct gaseous connection between the outside of the body and the airways. This means that the lungs are incorrectly removed by the region growing algorithm and are not included in the gas visualization. Second, because the algorithm generates multiple images that are superimposed, findings can be omitted. This may be the covering of a gas embolism by a physiological air-filled lung or a medical implant covering up a bone fracture. This means, the structures visualized using the MIP should not be used to assess bone fractures, but rather as a visual aid to give anatomical context. In cases where fracture patterns have to be visualized, volume rendering techniques are better suited [1, 6]. For a detailed radiological assessment of gas distributions, conventional 2D reading of the images should be performed. Third, because the projection is orthographic, the size of an object in the image cannot be used to determine the position of the object in the 3D dataset. The actual position of a finding on an anterior–posterior reconstruction can only be determined with a second, perpendicular reconstruction. Fourth, gas accumulations and foreign bodies that are below the resolution of the CT scanner may not be visualized because of an alteration in the HU due to partial volume effects.

In summary, the images cannot and should not be used to replace radiological assessment of the full CT dataset but rather allow for a quick overview of bones, gas distribution and foreign bodies. This plays an important role in decision making at earlier stages of a case and allows quick conveyance of these messages to medical laypeople involved in the case. Automated visualizations such as the one presented in this paper may help in streamlining briefings and help in the automatic generation of image reports.

## Key points

1. A combination of different imaging techniques is used to generate overview images. These overview images depict foreign bodies and gas accumulations in their anatomical context.
2. The algorithm works fully automatically without any user interaction.
3. The automatically generated overview images from PMCT can help in decision making.
4. The automatically generated overview images can help in communicating findings to medical laypeople.
